# 4-{[2-(2,4-Dinitro­phen­yl)hydrazinyl­idene](phen­yl)meth­yl}-5-methyl-2-phenyl-1*H*-pyrazol-3(2*H*)-one ethanol monosolvate

**DOI:** 10.1107/S1600536812044935

**Published:** 2012-11-17

**Authors:** Omoruyi G. Idemudia, Alexander P. Sadimenko, Eric C. Hosten

**Affiliations:** a University of Fort Hare, Department of Chemistry, Private Bag X1314, Alice 5700, South Africa; b Nelson Mandela Metropolitan University, Department of Chemistry, PO Box 77000, Port Elizabeth 6031, South Africa

## Abstract

In the title compound, C_23_H_18_N_6_O_5_·C_2_H_6_O, all three benzene rings lie in an approximate plane [maximum deviation = 0.2688 (16) Å] that makes an angle of 53.56 (3)° with the plane of the pyrazolone ring. Intra­molecular N—H⋯O hydrogen bonds occur. In the crystal, the ethanol solvent mol­ecule links adjacent mol­ecules through N—H⋯O—H⋯O hydrogen bonds, leading to an infinite chain along the *c*-axis direction. The ethyl group of the ethanol solvent mol­ecule is disordered over two set of sites in a 0.762 (5):0.238 (5) ratio.

## Related literature

For a related structure, see: Idemudia *et al.* (2012 ▶).
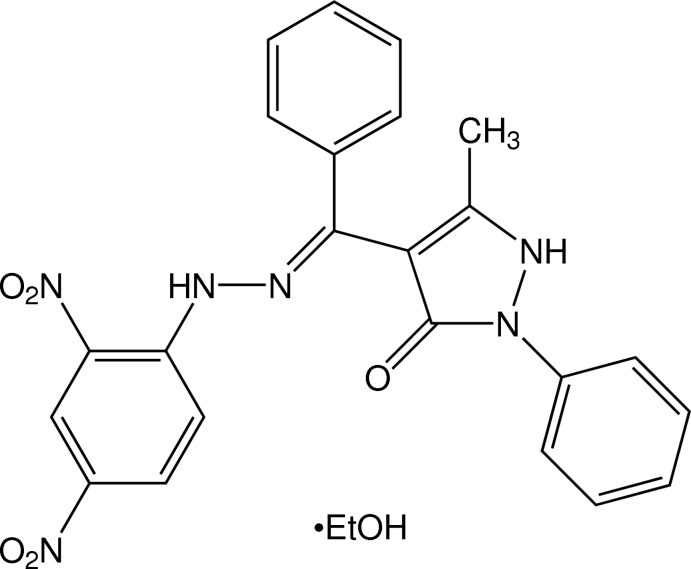


## Experimental

### 

#### Crystal data

C_23_H_18_N_6_O_5_·C_2_H_6_O
*M* *_r_* = 504.50
Monoclinic, 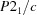

*a* = 12.8289 (4) Å
*b* = 14.3247 (4) Å
*c* = 14.4213 (4) Å
β = 111.347 (1)°
*V* = 2468.38 (12) Å^3^
*Z* = 4
Mo *K*α radiation
μ = 0.10 mm^−1^
*T* = 200 K
0.61 × 0.43 × 0.39 mm

#### Data collection

Bruker APEXII CCD diffractometer
Absorption correction: numerical (*SADABS*; Bruker, 2008 ▶) *T*_min_ = 0.89, *T*_max_ = 0.96
23873 measured reflections
6124 independent reflections
5136 reflections with *I* > 2σ(*I*)
*R*_int_ = 0.014

#### Refinement

*R*[*F* ^2^ > 2σ(*F* ^2^)] = 0.042
*wR*(*F* ^2^) = 0.116
*S* = 1.04
6124 reflections
368 parameters
H atoms treated by a mixture of independent and constrained refinement
Δρ_max_ = 0.28 e Å^−3^
Δρ_min_ = −0.22 e Å^−3^

### 

Data collection: *APEX2* (Bruker, 2010 ▶); cell refinement: *SAINT* (Bruker, 2010 ▶); data reduction: *SAINT*; program(s) used to solve structure: *SHELXS97* (Sheldrick, 2008 ▶); program(s) used to refine structure: *SHELXL97* (Sheldrick, 2008 ▶) and *SHELXLE* (Hübschle *et al.*, 2011 ▶); molecular graphics: *ORTEP-3* (Farrugia, 1997 ▶); software used to prepare material for publication: *PLATON* (Spek, 2009 ▶) and *publCIF* (Westrip, 2010 ▶).

## Supplementary Material

Crystal structure: contains datablock(s) global, I. DOI: 10.1107/S1600536812044935/ng5294sup1.cif

Structure factors: contains datablock(s) I. DOI: 10.1107/S1600536812044935/ng5294Isup2.hkl

Supplementary material file. DOI: 10.1107/S1600536812044935/ng5294Isup3.cdx

Supplementary material file. DOI: 10.1107/S1600536812044935/ng5294Isup4.cml

Additional supplementary materials:  crystallographic information; 3D view; checkCIF report

## Figures and Tables

**Table 1:** Hydrogen-bond geometry (Å, °)

*D*—H⋯*A*	*D*—H	H⋯*A*	*D*⋯*A*	*D*—H⋯*A*
N4—H4*N*⋯O1	0.915 (17)	2.023 (17)	2.8261 (13)	145.6 (14)
N4—H4*N*⋯O2	0.915 (17)	2.040 (17)	2.6497 (14)	122.8 (13)
N2—H2*N*⋯O6^i^	0.936 (18)	1.711 (18)	2.6464 (14)	177.4 (16)
O6—H6⋯O1	0.89 (2)	1.72 (2)	2.5863 (14)	167 (2)

Symmetry code: (i) 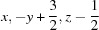.

**Fig. 1.**
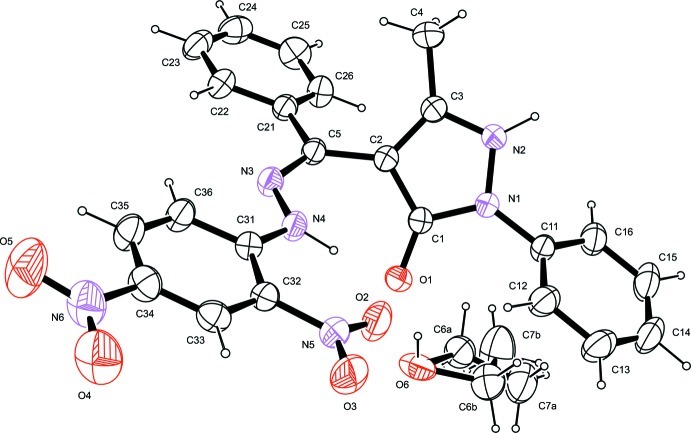
Molecular structure of the titled compound, with atom labels and displacement ellipsoids drawn at 50% probability level.

**Fig. 2.**
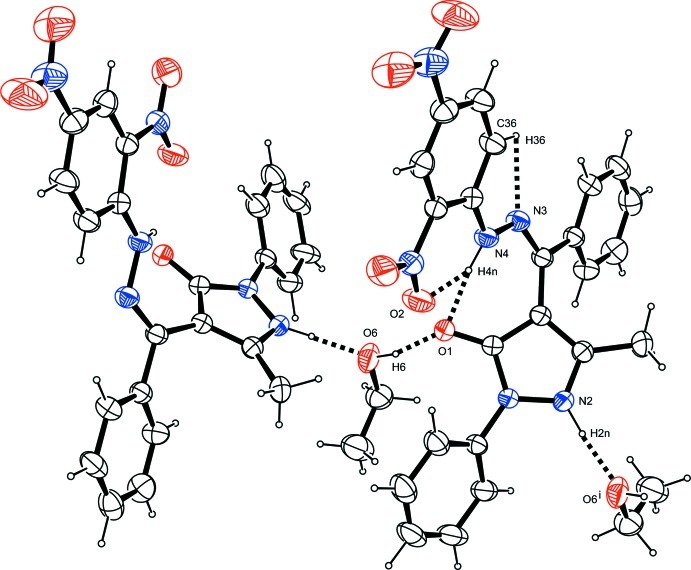
Selected intra and inter molecular contacts, with displacement ellipsoids drawn at 50% probability level. Symmetry operators: ix, -y + 3/2, z - 1/2.

## supplementary crystallographic information

### Comment

Acylpyrazolone derived heterocycles have received considerable research interest due to their versatile reactivity and enormous applications. So have phenylhydrazine Schiff bases and its derivatives. We have reported the crystal structure of a phenyl hydrazone with 4-benzoyl-3-methyl-1-phenyl-2-pyrazolin-5-one (Idemudia *et al.*, 2012). Presented herein is the crystal structure report of an acylpyrazolone based dinitrophenyl hydrazone in continuation of our probe on acylpyrazolone Schiff bases.

In the titled compound the least square plane of the pyrazolone ring makes a dihedral angle of 53.56 (3) ° with the least square plane of all the phenyl rings C11–C16, C21–C26, and C31–C36. The largest deviation distance from the phenyl ring plane is C14 at 0.2688 (16) Å. The dihedral angles with the individual least square planes through the phenyl rings with the pyrazolone plane are 45.87 (7) °, 58.29 (7) ° and 55.84 (7) ° repectively. The ethyl group of the ethanol solvent molecule is disordered over two set of sites [occupancy ratio 0.762 (5):0.238 (5)].

H4N, the hydrogen on N4, has two intra molecular contacts of 2.023 (17) Å and 2.040 (17) Å with O1 and O2 respectievly (Table 1). A C36—H36···N3 intra molecular contact of 2.34 Å also occurs. Molecules of the title compound are stacked in the *c* axis direction and linked *via* the ethanol solvent molecule with N2—H2N···O6 and O6—H6···O1 inter molecular contacts of length 1.711 (18) Å and 1.72 (2) Å respectively (Figure 2). Adjacent molecules have a C26—H26···O5 interaction of 2.43 Å (Table 1).

### Experimental

An equimolar mixture of 2,4-dinitrophenyl hydrazine and 4-benzoyl-3-methyl-1-phenyl-2-pyrazoline-5-one in ethanol stirred under reflux for 4 h, afforded a precipitate of the titled compound. Slow evaporation at room temperature of an ethanolic solution of it, gave red single crystals suitable for X-ray diffraction with a melting point of 233–236 °C after a few days.

### Refinement

Carbon bound H atoms were placed in calculated positions and refined as riding atoms, with C—H = 0.95 Å (aromatic CH), 0.99 Å (CH_2_), 0.98 Å (CH_3_) and with *U*_iso_(H) = 1.2 (1.5 for methyl) *U*_eq_(C). The nitrogen and oxygen bound H atoms were located on a difference Fourier map and allowed to refine freely. The reflections 011 and 100 were omitted from the refinement since they were obscured by the beam-stop.

### Crystal data

**Table:**

C_23_H_18_N_6_O_5_·C_2_H_6_O	*D*_x_ = 1.358 Mg m^−^^3^
*M**_r_* = 504.50	Melting point: 507 K
Monoclinic, *P*2_1_/*c*	Mo *K*α radiation, λ = 0.71073 Å
*a* = 12.8289 (4) Å	Cell parameters from 94 reflections
*b* = 14.3247 (4) Å	θ = 2.9–28.9°
*c* = 14.4213 (4) Å	µ = 0.10 mm^−^^1^
β = 111.347 (1)°	*T* = 200 K
*V* = 2468.38 (12) Å^3^	Block, red
*Z* = 4	0.61 × 0.43 × 0.39 mm
*F*(000) = 1056	

### Data collection

**Table:**

Bruker APEXII CCD diffractometer	6124 independent reflections
Graphite monochromator	5136 reflections with *I* > 2σ(*I*)
Detector resolution: 8.3333 pixels mm^-1^	*R*_int_ = 0.014
φ and ω scans	θ_max_ = 28.3°, θ_min_ = 2.2°
Absorption correction: numerical (*SADABS*; Bruker, 2008)	*h* = −16→17
*T*_min_ = 0.89, *T*_max_ = 0.96	*k* = −13→19
23873 measured reflections	*l* = −19→19

### Refinement

**Table:**

Refinement on *F*^2^	Primary atom site location: structure-invariant direct methods
Least-squares matrix: full	Secondary atom site location: difference Fourier map
*R*[*F*^2^ > 2σ(*F*^2^)] = 0.042	Hydrogen site location: inferred from neighbouring sites
*wR*(*F*^2^) = 0.116	H atoms treated by a mixture of independent and constrained refinement
*S* = 1.04	*w* = 1/[σ^2^(*F*_o_^2^) + (0.0507*P*)^2^ + 0.8241*P*] where *P* = (*F*_o_^2^ + 2*F*_c_^2^)/3
6124 reflections	(Δ/σ)_max_ < 0.001
368 parameters	Δρ_max_ = 0.28 e Å^−^^3^
0 restraints	Δρ_min_ = −0.22 e Å^−^^3^

### Special details

**Table:**

Geometry. All e.s.d.'s (except the e.s.d. in the dihedral angle between two l.s. planes) are estimated using the full covariance matrix. The cell e.s.d.'s are taken into account individually in the estimation of e.s.d.'s in distances, angles and torsion angles; correlations between e.s.d.'s in cell parameters are only used when they are defined by crystal symmetry. An approximate (isotropic) treatment of cell e.s.d.'s is used for estimating e.s.d.'s involving l.s. planes.
Refinement. Refinement of *F*^2^ against ALL reflections. The weighted *R*-factor *wR* and goodness of fit *S* are based on *F*^2^, conventional *R*-factors *R* are based on *F*, with *F* set to zero for negative *F*^2^. The threshold expression of *F*^2^ > σ(*F*^2^) is used only for calculating *R*-factors(gt) *etc*. and is not relevant to the choice of reflections for refinement. *R*-factors based on *F*^2^ are statistically about twice as large as those based on *F*, and *R*- factors based on ALL data will be even larger.

### Fractional atomic coordinates and isotropic or equivalent isotropic displacement parameters (Å^2^)

**Table:**

	*x*	*y*	*z*	*U*_iso_*/*U*_eq_	Occ. (<1)
O1	0.14776 (8)	0.69594 (6)	0.47229 (6)	0.0383 (2)	
O2	−0.07049 (9)	0.77842 (7)	0.42852 (9)	0.0543 (3)	
O3	−0.22537 (11)	0.83873 (7)	0.42516 (10)	0.0588 (3)	
O4	−0.53149 (10)	0.65498 (10)	0.42226 (12)	0.0730 (4)	
O5	−0.53782 (10)	0.50904 (11)	0.38432 (14)	0.0876 (5)	
O6	0.30397 (9)	0.73560 (9)	0.64059 (8)	0.0517 (3)	
N1	0.20431 (9)	0.73686 (7)	0.34187 (7)	0.0317 (2)	
N2	0.20240 (9)	0.69518 (7)	0.25557 (8)	0.0341 (2)	
N3	−0.01147 (8)	0.51321 (7)	0.38324 (8)	0.0325 (2)	
N4	−0.05711 (9)	0.59824 (7)	0.39281 (8)	0.0328 (2)	
N5	−0.16820 (10)	0.77102 (7)	0.42199 (8)	0.0399 (2)	
N6	−0.48924 (10)	0.58418 (10)	0.40330 (11)	0.0553 (3)	
C1	0.15772 (9)	0.67707 (8)	0.39078 (8)	0.0299 (2)	
C2	0.12604 (9)	0.59567 (8)	0.33014 (8)	0.0294 (2)	
C3	0.15677 (10)	0.61039 (8)	0.24838 (9)	0.0321 (2)	
C4	0.14119 (14)	0.55220 (10)	0.15841 (10)	0.0466 (3)	
H4A	0.1162	0.5919	0.099	0.07*	
H4B	0.0848	0.504	0.1522	0.07*	
H4C	0.2123	0.5225	0.1649	0.07*	
C5	0.07256 (9)	0.51282 (8)	0.35381 (8)	0.0292 (2)	
C11	0.24261 (10)	0.82994 (8)	0.36714 (9)	0.0322 (2)	
C12	0.17815 (12)	0.89102 (9)	0.39821 (11)	0.0424 (3)	
H12	0.1083	0.8716	0.4005	0.051*	
C13	0.21730 (15)	0.98106 (10)	0.42595 (12)	0.0514 (4)	
H13	0.1746	1.0234	0.4484	0.062*	
C14	0.31808 (15)	1.00912 (10)	0.42101 (12)	0.0532 (4)	
H14	0.3447	1.0706	0.4406	0.064*	
C15	0.38033 (14)	0.94861 (11)	0.38782 (12)	0.0520 (4)	
H15	0.4485	0.9691	0.3828	0.062*	
C16	0.34373 (11)	0.85755 (9)	0.36157 (10)	0.0410 (3)	
H16	0.3873	0.8151	0.3402	0.049*	
C21	0.11659 (10)	0.41870 (8)	0.34431 (8)	0.0304 (2)	
C22	0.05139 (11)	0.33871 (9)	0.33661 (10)	0.0375 (3)	
H22	−0.0221	0.3442	0.3374	0.045*	
C23	0.09362 (13)	0.25170 (9)	0.32782 (11)	0.0453 (3)	
H23	0.0489	0.1977	0.3227	0.054*	
C24	0.20034 (13)	0.24258 (10)	0.32641 (10)	0.0461 (3)	
H24	0.2282	0.1827	0.3189	0.055*	
C25	0.26619 (12)	0.32073 (10)	0.33591 (11)	0.0455 (3)	
H25	0.34	0.3147	0.3361	0.055*	
C26	0.22459 (11)	0.40835 (9)	0.34526 (10)	0.0379 (3)	
H26	0.2706	0.4618	0.3524	0.045*	
C31	−0.16063 (10)	0.59744 (8)	0.39772 (8)	0.0302 (2)	
C32	−0.21772 (10)	0.67863 (8)	0.41052 (9)	0.0322 (2)	
C33	−0.32476 (11)	0.67409 (9)	0.41267 (9)	0.0364 (3)	
H33	−0.3614	0.7291	0.4215	0.044*	
C34	−0.37698 (10)	0.58920 (10)	0.40188 (10)	0.0393 (3)	
C35	−0.32465 (11)	0.50752 (10)	0.38895 (11)	0.0422 (3)	
H35	−0.3622	0.4493	0.3817	0.051*	
C36	−0.21948 (11)	0.51165 (9)	0.38672 (10)	0.0378 (3)	
H36	−0.1846	0.4557	0.3776	0.045*	
H2N	0.2371 (14)	0.7212 (12)	0.2146 (13)	0.054 (5)*	
H4N	−0.0119 (14)	0.6495 (12)	0.4120 (12)	0.050 (4)*	
H6	0.2561 (18)	0.7277 (14)	0.5787 (17)	0.072 (6)*	
C6A	0.41626 (18)	0.74427 (15)	0.64338 (17)	0.0497 (6)	0.762 (5)
H6AA	0.4222	0.7136	0.584	0.06*	0.762 (5)
H6AB	0.4678	0.7119	0.7031	0.06*	0.762 (5)
C7A	0.4500 (3)	0.8432 (2)	0.64576 (19)	0.0669 (9)	0.762 (5)
H7AA	0.4044	0.8737	0.5833	0.1*	0.762 (5)
H7AB	0.5292	0.8467	0.6539	0.1*	0.762 (5)
H7AC	0.4389	0.8749	0.7017	0.1*	0.762 (5)
C6B	0.3762 (6)	0.8194 (5)	0.6360 (5)	0.053 (2)	0.238 (5)
H6BA	0.3433	0.8529	0.5719	0.064*	0.238 (5)
H6BB	0.3852	0.8636	0.6913	0.064*	0.238 (5)
C7B	0.4832 (8)	0.7761 (8)	0.6459 (10)	0.091 (4)	0.238 (5)
H7BA	0.4727	0.7356	0.5885	0.136*	0.238 (5)
H7BB	0.5105	0.7389	0.7071	0.136*	0.238 (5)
H7BC	0.5379	0.8248	0.6488	0.136*	0.238 (5)

### Atomic displacement parameters (Å^2^)

**Table:**

	*U*^11^	*U*^22^	*U*^33^	*U*^12^	*U*^13^	*U*^23^
O1	0.0441 (5)	0.0430 (5)	0.0328 (4)	−0.0148 (4)	0.0199 (4)	−0.0091 (4)
O2	0.0559 (6)	0.0345 (5)	0.0824 (8)	−0.0116 (4)	0.0368 (6)	−0.0076 (5)
O3	0.0760 (8)	0.0300 (5)	0.0843 (8)	0.0055 (5)	0.0457 (7)	−0.0046 (5)
O4	0.0488 (6)	0.0734 (8)	0.1089 (11)	0.0175 (6)	0.0432 (7)	0.0078 (8)
O5	0.0437 (7)	0.0796 (10)	0.1479 (15)	−0.0183 (6)	0.0448 (8)	−0.0171 (9)
O6	0.0405 (5)	0.0805 (8)	0.0385 (5)	−0.0101 (5)	0.0197 (4)	−0.0182 (5)
N1	0.0401 (5)	0.0269 (5)	0.0327 (5)	−0.0068 (4)	0.0186 (4)	−0.0038 (4)
N2	0.0466 (6)	0.0290 (5)	0.0335 (5)	−0.0046 (4)	0.0229 (4)	−0.0024 (4)
N3	0.0329 (5)	0.0267 (5)	0.0399 (5)	−0.0011 (4)	0.0154 (4)	−0.0001 (4)
N4	0.0347 (5)	0.0252 (5)	0.0423 (5)	−0.0032 (4)	0.0185 (4)	−0.0012 (4)
N5	0.0526 (7)	0.0289 (5)	0.0443 (6)	−0.0025 (5)	0.0249 (5)	−0.0027 (4)
N6	0.0333 (6)	0.0630 (9)	0.0725 (9)	0.0017 (6)	0.0227 (6)	0.0030 (7)
C1	0.0308 (5)	0.0297 (5)	0.0305 (5)	−0.0050 (4)	0.0129 (4)	−0.0017 (4)
C2	0.0323 (5)	0.0259 (5)	0.0314 (5)	−0.0024 (4)	0.0134 (4)	−0.0012 (4)
C3	0.0383 (6)	0.0267 (5)	0.0338 (6)	−0.0006 (4)	0.0162 (5)	−0.0015 (4)
C4	0.0689 (9)	0.0375 (7)	0.0414 (7)	−0.0057 (6)	0.0297 (7)	−0.0093 (5)
C5	0.0296 (5)	0.0269 (5)	0.0302 (5)	−0.0041 (4)	0.0098 (4)	−0.0006 (4)
C11	0.0394 (6)	0.0254 (5)	0.0311 (5)	−0.0054 (4)	0.0119 (5)	−0.0003 (4)
C12	0.0466 (7)	0.0338 (6)	0.0478 (7)	−0.0022 (5)	0.0182 (6)	−0.0054 (5)
C13	0.0679 (10)	0.0303 (7)	0.0527 (8)	0.0016 (6)	0.0181 (7)	−0.0058 (6)
C14	0.0717 (10)	0.0267 (6)	0.0499 (8)	−0.0124 (6)	0.0089 (7)	0.0011 (6)
C15	0.0540 (8)	0.0406 (8)	0.0571 (9)	−0.0187 (7)	0.0151 (7)	0.0049 (6)
C16	0.0425 (7)	0.0354 (6)	0.0466 (7)	−0.0072 (5)	0.0181 (6)	0.0018 (5)
C21	0.0328 (6)	0.0273 (5)	0.0298 (5)	−0.0020 (4)	0.0098 (4)	−0.0001 (4)
C22	0.0366 (6)	0.0318 (6)	0.0414 (6)	−0.0067 (5)	0.0108 (5)	−0.0041 (5)
C23	0.0542 (8)	0.0294 (6)	0.0461 (7)	−0.0078 (6)	0.0110 (6)	−0.0064 (5)
C24	0.0596 (9)	0.0319 (6)	0.0423 (7)	0.0098 (6)	0.0132 (6)	−0.0015 (5)
C25	0.0431 (7)	0.0435 (7)	0.0509 (8)	0.0108 (6)	0.0184 (6)	0.0043 (6)
C26	0.0348 (6)	0.0330 (6)	0.0465 (7)	−0.0002 (5)	0.0155 (5)	0.0034 (5)
C31	0.0317 (5)	0.0293 (5)	0.0306 (5)	−0.0022 (4)	0.0124 (4)	−0.0004 (4)
C32	0.0384 (6)	0.0280 (6)	0.0317 (5)	−0.0011 (5)	0.0145 (5)	−0.0002 (4)
C33	0.0375 (6)	0.0374 (6)	0.0354 (6)	0.0061 (5)	0.0147 (5)	0.0016 (5)
C34	0.0284 (6)	0.0458 (7)	0.0442 (7)	0.0004 (5)	0.0137 (5)	0.0007 (5)
C35	0.0341 (6)	0.0367 (7)	0.0555 (8)	−0.0072 (5)	0.0159 (6)	−0.0049 (6)
C36	0.0342 (6)	0.0295 (6)	0.0511 (7)	−0.0033 (5)	0.0172 (5)	−0.0050 (5)
C6A	0.0436 (12)	0.0495 (12)	0.0643 (13)	−0.0020 (9)	0.0296 (9)	−0.0051 (9)
C7A	0.072 (2)	0.0563 (16)	0.0620 (14)	−0.0192 (13)	0.0121 (12)	0.0037 (11)
C6B	0.044 (4)	0.060 (5)	0.049 (4)	−0.006 (3)	0.008 (3)	0.001 (3)
C7B	0.052 (5)	0.074 (7)	0.150 (10)	0.005 (4)	0.041 (6)	0.025 (6)

### Geometric parameters (Å, º)

**Table:**

O1—C1	1.2572 (14)	C15—C16	1.3913 (19)
O2—N5	1.2270 (15)	C15—H15	0.95
O3—N5	1.2269 (15)	C16—H16	0.95
O4—N6	1.2268 (18)	C21—C26	1.3886 (17)
O5—N6	1.2238 (19)	C21—C22	1.3991 (16)
O6—C6A	1.432 (2)	C22—C23	1.3831 (19)
O6—C6B	1.533 (8)	C22—H22	0.95
O6—H6	0.89 (2)	C23—C24	1.383 (2)
N1—N2	1.3725 (13)	C23—H23	0.95
N1—C1	1.3767 (14)	C24—C25	1.379 (2)
N1—C11	1.4218 (14)	C24—H24	0.95
N2—C3	1.3358 (15)	C25—C26	1.3895 (18)
N2—H2N	0.936 (18)	C25—H25	0.95
N3—C5	1.2942 (15)	C26—H26	0.95
N3—N4	1.3801 (14)	C31—C36	1.4206 (16)
N4—C31	1.3554 (15)	C31—C32	1.4219 (16)
N4—H4N	0.915 (17)	C32—C33	1.3864 (17)
N5—C32	1.4512 (15)	C33—C34	1.3696 (19)
N6—C34	1.4496 (17)	C33—H33	0.95
C1—C2	1.4251 (15)	C34—C35	1.3948 (19)
C2—C3	1.3888 (16)	C35—C36	1.3626 (18)
C2—C5	1.4718 (15)	C35—H35	0.95
C3—C4	1.4931 (17)	C36—H36	0.95
C4—H4A	0.98	C6A—C7A	1.479 (3)
C4—H4B	0.98	C6A—H6AA	0.99
C4—H4C	0.98	C6A—H6AB	0.99
C5—C21	1.4873 (16)	C7A—H7AA	0.98
C11—C12	1.3856 (18)	C7A—H7AB	0.98
C11—C16	1.3863 (18)	C7A—H7AC	0.98
C12—C13	1.3891 (19)	C6B—C7B	1.466 (13)
C12—H12	0.95	C6B—H6BA	0.99
C13—C14	1.380 (2)	C6B—H6BB	0.99
C13—H13	0.95	C7B—H7BA	0.98
C14—C15	1.377 (2)	C7B—H7BB	0.98
C14—H14	0.95	C7B—H7BC	0.98
			
C6A—O6—H6	111.1 (13)	C26—C21—C22	118.61 (11)
C6B—O6—H6	105.4 (13)	C26—C21—C5	120.47 (10)
N2—N1—C1	109.04 (9)	C22—C21—C5	120.91 (11)
N2—N1—C11	122.07 (9)	C23—C22—C21	120.16 (12)
C1—N1—C11	128.75 (10)	C23—C22—H22	119.9
C3—N2—N1	109.19 (9)	C21—C22—H22	119.9
C3—N2—H2N	127.5 (11)	C24—C23—C22	120.63 (13)
N1—N2—H2N	122.9 (11)	C24—C23—H23	119.7
C5—N3—N4	118.09 (10)	C22—C23—H23	119.7
C31—N4—N3	117.20 (10)	C25—C24—C23	119.72 (13)
C31—N4—H4N	121.1 (10)	C25—C24—H24	120.1
N3—N4—H4N	119.6 (10)	C23—C24—H24	120.1
O3—N5—O2	122.46 (11)	C24—C25—C26	120.00 (13)
O3—N5—C32	118.84 (11)	C24—C25—H25	120.0
O2—N5—C32	118.70 (10)	C26—C25—H25	120.0
O5—N6—O4	123.14 (13)	C21—C26—C25	120.84 (12)
O5—N6—C34	117.91 (13)	C21—C26—H26	119.6
O4—N6—C34	118.95 (13)	C25—C26—H26	119.6
O1—C1—N1	123.79 (10)	N4—C31—C36	119.51 (11)
O1—C1—C2	130.26 (10)	N4—C31—C32	124.10 (10)
N1—C1—C2	105.94 (9)	C36—C31—C32	116.37 (10)
C3—C2—C1	106.92 (10)	C33—C32—C31	121.72 (11)
C3—C2—C5	128.31 (10)	C33—C32—N5	115.87 (11)
C1—C2—C5	124.77 (10)	C31—C32—N5	122.41 (11)
N2—C3—C2	108.91 (10)	C34—C33—C32	119.12 (11)
N2—C3—C4	119.43 (11)	C34—C33—H33	120.4
C2—C3—C4	131.56 (11)	C32—C33—H33	120.4
C3—C4—H4A	109.5	C33—C34—C35	121.37 (11)
C3—C4—H4B	109.5	C33—C34—N6	119.33 (12)
H4A—C4—H4B	109.5	C35—C34—N6	119.30 (12)
C3—C4—H4C	109.5	C36—C35—C34	119.71 (12)
H4A—C4—H4C	109.5	C36—C35—H35	120.1
H4B—C4—H4C	109.5	C34—C35—H35	120.1
N3—C5—C2	125.94 (10)	C35—C36—C31	121.70 (12)
N3—C5—C21	115.07 (10)	C35—C36—H36	119.1
C2—C5—C21	119.00 (10)	C31—C36—H36	119.1
C12—C11—C16	121.32 (12)	O6—C6A—C7A	111.5 (2)
C12—C11—N1	118.94 (11)	O6—C6A—H6AA	109.3
C16—C11—N1	119.74 (11)	C7A—C6A—H6AA	109.3
C11—C12—C13	118.93 (13)	O6—C6A—H6AB	109.3
C11—C12—H12	120.5	C7A—C6A—H6AB	109.3
C13—C12—H12	120.5	H6AA—C6A—H6AB	108.0
C14—C13—C12	120.18 (14)	C7B—C6B—O6	102.9 (6)
C14—C13—H13	119.9	C7B—C6B—H6BA	111.2
C12—C13—H13	119.9	O6—C6B—H6BA	111.2
C15—C14—C13	120.48 (13)	C7B—C6B—H6BB	111.2
C15—C14—H14	119.8	O6—C6B—H6BB	111.2
C13—C14—H14	119.8	H6BA—C6B—H6BB	109.1
C14—C15—C16	120.25 (14)	C6B—C7B—H7BA	109.5
C14—C15—H15	119.9	C6B—C7B—H7BB	109.5
C16—C15—H15	119.9	H7BA—C7B—H7BB	109.5
C11—C16—C15	118.82 (13)	C6B—C7B—H7BC	109.5
C11—C16—H16	120.6	H7BA—C7B—H7BC	109.5
C15—C16—H16	120.6	H7BB—C7B—H7BC	109.5
			
C1—N1—N2—C3	−0.45 (14)	N3—C5—C21—C26	−159.14 (11)
C11—N1—N2—C3	−176.44 (11)	C2—C5—C21—C26	20.72 (16)
C5—N3—N4—C31	163.07 (11)	N3—C5—C21—C22	19.34 (16)
N2—N1—C1—O1	−179.97 (11)	C2—C5—C21—C22	−160.80 (11)
C11—N1—C1—O1	−4.3 (2)	C26—C21—C22—C23	−1.49 (19)
N2—N1—C1—C2	−0.23 (13)	C5—C21—C22—C23	180.00 (12)
C11—N1—C1—C2	175.41 (11)	C21—C22—C23—C24	−0.1 (2)
O1—C1—C2—C3	−179.48 (13)	C22—C23—C24—C25	1.3 (2)
N1—C1—C2—C3	0.80 (13)	C23—C24—C25—C26	−1.0 (2)
O1—C1—C2—C5	−0.3 (2)	C22—C21—C26—C25	1.82 (19)
N1—C1—C2—C5	179.97 (11)	C5—C21—C26—C25	−179.67 (12)
N1—N2—C3—C2	0.97 (14)	C24—C25—C26—C21	−0.6 (2)
N1—N2—C3—C4	177.64 (12)	N3—N4—C31—C36	−3.89 (17)
C1—C2—C3—N2	−1.10 (14)	N3—N4—C31—C32	178.08 (11)
C5—C2—C3—N2	179.77 (11)	N4—C31—C32—C33	178.43 (11)
C1—C2—C3—C4	−177.22 (14)	C36—C31—C32—C33	0.34 (17)
C5—C2—C3—C4	3.6 (2)	N4—C31—C32—N5	−1.50 (18)
N4—N3—C5—C2	0.72 (17)	C36—C31—C32—N5	−179.59 (11)
N4—N3—C5—C21	−179.44 (10)	O3—N5—C32—C33	−5.39 (17)
C3—C2—C5—N3	−134.04 (14)	O2—N5—C32—C33	174.35 (12)
C1—C2—C5—N3	46.97 (18)	O3—N5—C32—C31	174.54 (12)
C3—C2—C5—C21	46.12 (17)	O2—N5—C32—C31	−5.72 (18)
C1—C2—C5—C21	−132.87 (12)	C31—C32—C33—C34	−0.20 (18)
N2—N1—C11—C12	131.90 (13)	N5—C32—C33—C34	179.73 (11)
C1—N1—C11—C12	−43.23 (18)	C32—C33—C34—C35	0.1 (2)
N2—N1—C11—C16	−48.92 (17)	C32—C33—C34—N6	−179.53 (12)
C1—N1—C11—C16	135.95 (13)	O5—N6—C34—C33	173.35 (16)
C16—C11—C12—C13	−1.2 (2)	O4—N6—C34—C33	−5.9 (2)
N1—C11—C12—C13	177.96 (12)	O5—N6—C34—C35	−6.3 (2)
C11—C12—C13—C14	1.0 (2)	O4—N6—C34—C35	174.49 (15)
C12—C13—C14—C15	0.6 (2)	C33—C34—C35—C36	−0.1 (2)
C13—C14—C15—C16	−1.8 (2)	N6—C34—C35—C36	179.48 (13)
C12—C11—C16—C15	−0.1 (2)	C34—C35—C36—C31	0.3 (2)
N1—C11—C16—C15	−179.21 (12)	N4—C31—C36—C35	−178.57 (12)
C14—C15—C16—C11	1.6 (2)	C32—C31—C36—C35	−0.39 (19)

### Hydrogen-bond geometry (Å, º)

**Table:**

*D*—H···*A*	*D*—H	H···*A*	*D*···*A*	*D*—H···*A*
N4—H4*N*···O1	0.915 (17)	2.023 (17)	2.8261 (13)	145.6 (14)
N4—H4*N*···O2	0.915 (17)	2.040 (17)	2.6497 (14)	122.8 (13)
N2—H2*N*···O6^i^	0.936 (18)	1.711 (18)	2.6464 (14)	177.4 (16)
O6—H6···O1	0.89 (2)	1.72 (2)	2.5863 (14)	167 (2)

Symmetry code: (i) *x*, −*y*+3/2, *z*−1/2.
